# Successful treatment of vacuum sealing drainage combined with super tension-relieving suture for nonhealing wound of a patient with classical Ehlers-Danlos syndrome

**DOI:** 10.1016/j.jdcr.2026.04.006

**Published:** 2026-04-13

**Authors:** Keshen Qu, Yiping Sun, Fang Liu, Meng Xing, Peipei Han

**Affiliations:** a Department of Dermatology, The Second Affiliated Hospital of Shaanxi University of Chinese Medicine, Xianyang, China; b College of Clinical Medicine, Shaanxi University of Chinese Medicine, Xianyang, Shaanxi, China; c Department of Pathology, The Second Affiliated Hospital of Shaanxi University of Chinese Medicine, Xianyang, China; d Department of Dermatology, Shaanxi Provincial Hospital of Chinese Medicine, Xi’an, China

**Keywords:** classical Ehlers-Danlos syndrome, nonhealing wound, super tension-relieving suture, vacuum sealing drainage

## Introduction

Ehlers–Danlos syndromes (EDS) are heritable connective tissue disorders caused by pathogenic variants that impair collagen biosynthesis or structure. According to the 2017 International Classification, EDS comprises 13 clinical subtypes, defined by distinct phenotypes and underlying genetic variants.^1^ Classical EDS (cEDS) is one of the most prevalent subtypes and is usually caused by variants in COL5A1 or COL5A2, which encode type V collagen.^2^ Core clinical features include skin hyperextensibility, atrophic scarring with poor wound healing, and generalized joint hypermobility.^3^ Although these signs can support clinical recognition, many patients remain undiagnosed until complications arise, for example after surgery. In such situations, confirmation of cEDS ideally relies on molecular genetic testing, where available.

We report a 10-year-old boy in whom cEDS was suspected because of postoperative wound dehiscence after open reduction and internal fixation of a distal radial fracture. The diagnosis was supported by characteristic clinical features and histopathologic findings. This case highlights the need to consider underlying connective tissue disorders in children who present with unexpected surgical complications.

## Case report

A previously healthy 10-year-old boy fell from a bicycle onto his right hand, causing immediate severe pain and swelling. Radiographs obtained showed a right distal radial fracture (Supplementary Fig 1, *A-C*, available via Mendeley at https://doi.org/10.17632/xxfpjcsjfm.2). Later, open reduction and internal fixation was performed under general anesthesia, and the incision was closed with interrupted sutures. He received routine intravenous antibiotics and daily iodine dressings.

On the third postoperative day, wound dehiscence was observed. The sutures were removed, debridement was performed, and daily topical iodine was continued. Five days later, after granulation tissue appeared, the wound was re-sutured (Supplementary Fig 2, *A*, available via Mendeley at https://doi.org/10.17632/xxfpjcsjfm.2), but dehiscence recurred the next day.

**Fig 1:**
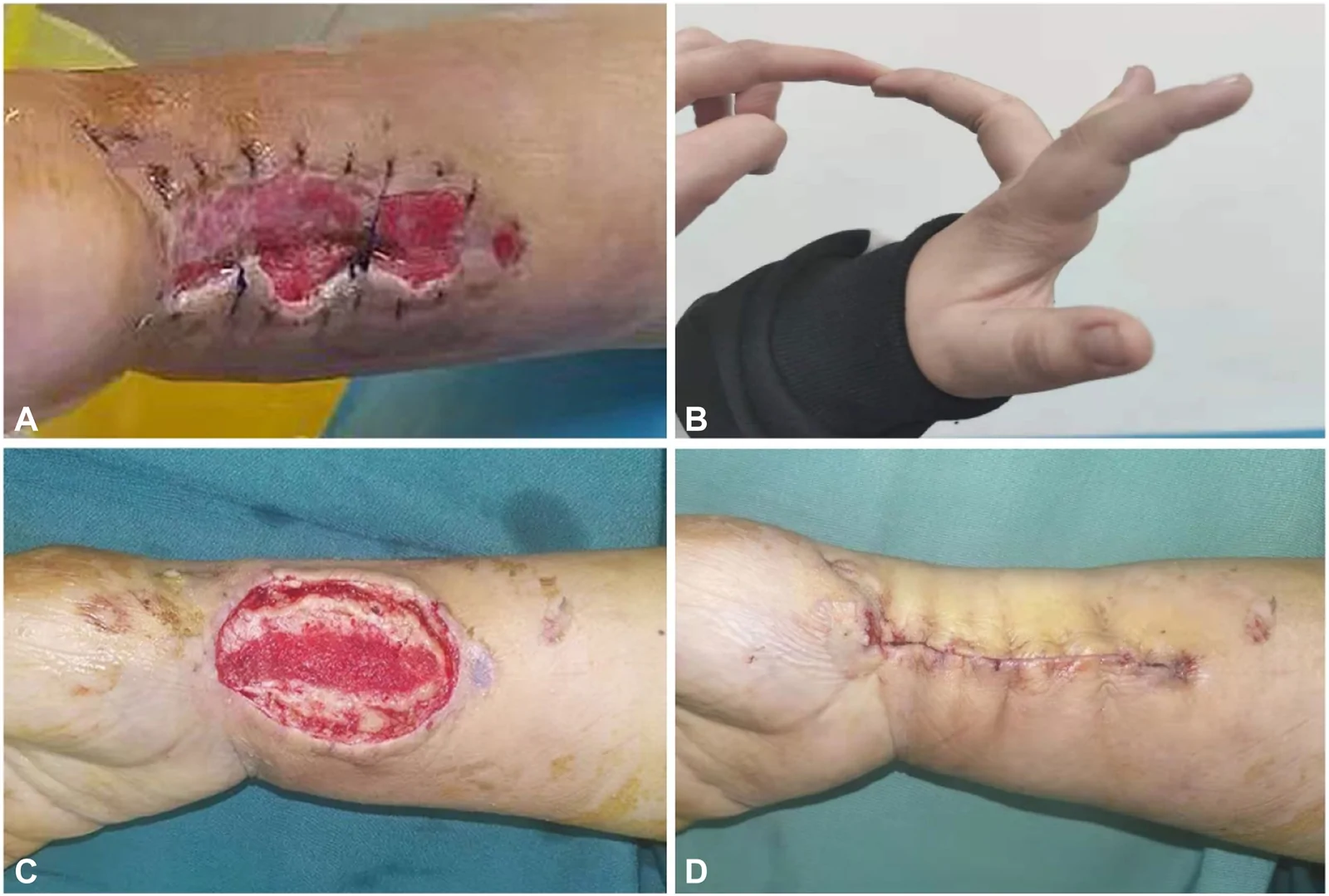
Clinical manifestations of typical Ehlers-Danlos syndrome patients. **A,** Sutured wound (7 × 2.5 × 1 cm) on the radial aspect of the right wrist showing superficial gray-white necrotic tissue and exudate, without surrounding erythema or swelling. **B,** Hypermobile phalangeal joints. **C,** Wound status after 7 days of VSD treatment when the device was removed. **D,** Final debridement performed, with successful wound closure using an intradermal butterfly suture technique. *VSD*, Vacuum sealing drainage.

The orthopedic team requested a dermatology consultation, and the patient was transferred for further evaluation. Apart from a history of minor trauma leading to ulceration, bleeding, and scarring, his past medical history was unremarkable. Examination revealed a cigarette paper–like atrophic scar on the right anterior tibia (Supplementary Fig 2, *C*, available via Mendeley at https://doi.org/10.17632/xxfpjcsjfm.2) and a 7 × 2.5 × 1 cm sutured wound on the radial wrist with superficial gray-white necrotic tissue but no erythema or swelling (Fig 1, A). The patient also had soft, doughy, hyperextensible skin and marked joint hypermobility of the hands, feet, wrists, fingers, and toes (Fig 1, B and Supplementary Fig 2, *B*, available via Mendeley at https://doi.org/10.17632/xxfpjcsjfm.2). His grandmother, great-grandmother, mother, and uncle reportedly had similar features. Recurrent wound dehiscence, typical skin and joint findings, and this multigenerational family history strongly suggested cEDS.

To manage the nonhealing wound and clarify the diagnosis, bacterial culture, repeat debridement, and a full-thickness skin biopsy from the wound edge were performed. Negative pressure wound therapy (NPWT), also known as vacuum sealing drainage (VSD), with continuous irrigation was then applied. After 7 days, a clean wound bed with healthy granulation tissue and no purulence or necrosis was observed (Fig 1, C). Final debridement was followed by layered closure using a super tension-relieving suture (STRS) technique (Fig 1, D). Postoperative care included daily iodine dressings and standard bandaging. The incision healed completely, and sutures were removed after 14 days (Supplementary Fig 2, *D*, available via Mendeley at https://doi.org/10.17632/xxfpjcsjfm.2).

Histopathologic examination showed reduced collagen with loose, disorganized fibers, and increased elastic fibers on Elastic Van Gieson staining (Supplementary Fig 3, *A-D*, available via Mendeley at https://doi.org/10.17632/xxfpjcsjfm.2). These findings, together with skin hyperextensibility, atrophic scarring, generalized joint hypermobility, soft doughy skin, and a positive family history, and fulfilled clinical criteria for cEDS.^1^ The family declined molecular genetic testing.

## Discussion

### Clinical and molecular underpinnings of cEDS

Classical Ehlers–Danlos syndrome (cEDS) is an autosomal dominant connective tissue disorder mainly caused by pathogenic variants in COL5A1 and COL5A2, which encode type V collagen.^4^ Type V collagen, a regulatory fibril-forming collagen widely distributed in connective tissues, is essential for extracellular matrix organization and tissue integrity.^5^ In cEDS, impaired wound healing has been associated with disorganized type V collagen, reduced dermal fibroblast migration, and transcriptome-level changes in extracellular matrix–related genes, indicating complex molecular dysregulation that has not yet been fully elucidated. These molecular abnormalities likely contribute to the pronounced surgical and wound-healing challenges observed in patients with cEDS.

### Surgical challenges and wound management strategies in cEDS

Surgical management of patients with cEDS is technically demanding because of tissue fragility, poor suture retention, and recurrent wound dehiscence. Case-based evidence suggests that tailored reconstructive approaches, can achieve satisfactory wound closure in selected patients.^6^^,^^7^ Nevertheless, robust clinical data and consensus guidelines on optimal perioperative and wound care protocols in cEDS are lacking, underscoring the need for systematic studies and standardized treatment strategies.^8^

In the present case, conventional closure techniques led to repeated wound dehiscence, emphasizing the importance of both biological and biomechanical factors in surgical planning for patients with suspected connective tissue disorders.

### Rationale for VSD and super tension-relieving suture (STRS)

Negative pressure wound therapy (NPWT/VSD) is an effective adjunct for the management of complex wounds. It maintains a closed moist environment, removes exudate, and stimulates granulation tissue formation, while modulating angiogenesis and inflammation to create a more favorable microenvironment for healing.^9^

In our patient, NPWT/VSD was used to optimize the wound bed by promoting granulation and controlling local bioburden before definitive closure. The STRS technique was then employed to achieve layered, low-tension closure by redistributing mechanical load from the epidermis to the deeper dermal and fascial layers. This dual strategy—addressing both biological impediments to healing and excess mechanical tension—was associated with durable wound closure and minimal scarring in this pediatric cEDS patient.^10^

A limitation of this report is the absence of molecular confirmation, as the family declined genetic testing. Nevertheless, the combination of characteristic clinical features, supportive histopathological findings, and a clear multigenerational family history provided strong evidence for a diagnosis of cEDS.

Although the favorable outcome in this case is encouraging, it remains uncertain whether similar results would be reproducible across EDS subtypes and wound contexts. Larger studies (eg, multicenter case series or prospective comparative cohorts) are needed to define safety and effectiveness.

## Conclusion

We propose that the combination of VSD and STRS may represent a viable therapeutic option for complex wound management in patients with cEDS. In this case, NPWT/VSD facilitated wound bed optimization, and STRS enabled durable, low-tension closure, together resulting in successful healing with minimal scarring. This approach should be considered during surgical planning for patients with known or suspected connective tissue disorders exhibiting tissue fragility.

## Conflicts of interest

None disclosed.
